# Phylogenomic and comparative analyses of Coffeeae alliance (Rubiaceae): deep insights into phylogenetic relationships and plastome evolution

**DOI:** 10.1186/s12870-022-03480-5

**Published:** 2022-02-26

**Authors:** Sara Getachew Amenu, Neng Wei, Lei Wu, Oyetola Oyebanji, Guangwan Hu, Yadong Zhou, Qingfeng Wang

**Affiliations:** 1 Key Laboratory of Plant Germplasm Enhancement and Specialty Agriculture, Wuhan Botanical Garden, Chinese Academy of Sciences, Wuhan, 430074 Hubei People’s Republic of China; 2 University of Chinese Academy of Sciences, Beijing, 100049 People’s Republic of China; 3 College of Forestry, Central South University of Forestry and Technology, Changsha, 410004 Hunan People’s Republic of China; 4 Germplasm Bank of Wild Species, Kunming Institute of Botany, Chinese Academy of Sciences, Kunming, 650201 Yunnan People’s Republic of China; 5 Department of Botany, Faculty of Science, University of Lagos, Lagos, Nigeria; 6 Center of Conservation Biology, Core Botanical Gardens, Chinese Academy of Sciences, Wuhan, 430074 Hubei People’s Republic of China; 7 Sino-Africa Joint Research Center (SAJOREC), Chinese Academy of Sciences, Wuhan, 430074 Hubei People’s Republic of China

**Keywords:** Coffeeae alliance, Phylogenomic, Plastome, Plastome structural variations (PSVs), Rubiaceae

## Abstract

**Background:**

The large and diverse Coffeeae alliance clade of subfamily Ixoroideae (Rubiaceae) consists of 10 tribes, > 90 genera, and > 2000 species. Previous molecular phylogenetics using limited numbers of markers were often unable to fully resolve the phylogenetic relationships at tribal and generic levels. Also, the structural variations of plastomes (PSVs) within the Coffeeae alliance tribes have been poorly investigated in previous studies. To fully understand the phylogenetic relationships and PSVs within the clade, highly reliable and sufficient sampling with superior next-generation analysis techniques is required. In this study, 71 plastomes (40 newly sequenced and assembled and the rest from the GenBank) were comparatively analyzed to decipher the PSVs and resolve the phylogenetic relationships of the Coffeeae alliance using four molecular data matrices.

**Results:**

All plastomes are typically quadripartite with the size ranging from 153,055 to 155,908 bp and contained 111 unique genes. The inverted repeat (IR) regions experienced multiple contraction and expansion; five repeat types were detected but the most abundant was SSR. The size of the Coffeeae alliance clade plastomes and its elements are affected by the IR boundary shifts and the repeat types. However, the emerging PSVs had no taxonomic and phylogenetic implications. Eight highly divergent regions were identified within the plastome regions *ndhF, ccsA, ndhD, ndhA, ndhH, ycf1*, *rps16-trnQ-UUG,* and *psbM-trnD*. These highly variable regions may be potential molecular markers for further species delimitation and population genetic analyses for the clade. Our plastome phylogenomic analyses yielded a well-resolved phylogeny tree with well-support at the tribal and generic levels within the Coffeeae alliance.

**Conclusions:**

Plastome data could be indispensable in resolving the phylogenetic relationships of the Coffeeae alliance tribes. Therefore, this study provides deep insights into the PSVs and phylogenetic relationships of the Coffeeae alliance and the Rubiaceae family as a whole.

**Supplementary Information:**

The online version contains supplementary material available at 10.1186/s12870-022-03480-5.

## Introduction

With over 13,000 species belonging to 611 genera and 65 tribes, Rubiaceae, commonly known as coffee, madder, or bedstraw family, is the fourth-largest angiosperm family after Asteraceae, Orchidaceae and Fabaceae [1]. Rubiaceae is cosmopolitan, occupies all the tropical and subtropical ecosystems [2]. The family occupies wide range of ecological niches from moist evergreen tropical lowland forests to deserts with altitudes 4000 m above sea level [3]. Also, the family is mostly characterized as tropical trees/shrubs and rarely annual/perennial herbs [4]; hence, often referred to as one of the most biomass-rich and species-rich woody plant families [3]. Despite the eminence of previous studies on the taxonomy of Rubiaceae, still the number of traditional subfamilies that should be recognized is yet to be fully substantiated [5, 6]. However, the recent classification [6] has suggested three subfamilies Rubioideae, Cinchonoideae, and Ixoroideae.

Subfamily Ixoroideae, the largest subfamily has the highest species richness which contain ca. 4000 species in 189 genera and 26 recognized tribes [7, 8]. The Ixoroideae is morphologically characterized by secondary pollen presentation, entire interpetiolar stipules, distorted aestivation, and various succulent fruits [9]. It also includes many well-known economically important genera used for horticultural e.g., *Gardenia* J.Ellis and *Ixora* L. [10] and medicine—e.g., *Ixora*., *Gardenia* and beverages *Coffe*a L. [11]. Broadly, two informal alliances are recognized within the Ixoroideae: the Vanguerieae alliance (8 tribes, > 37 genera and ca. 1200 species) and the Coffeeae alliance (10 tribes, > 90 genera and > 2000 species) [7, 8]. The Coffeeae alliance is an important radiation of the subfamily Ixoroideae and previous taxonomic classification was based on morphological features such as secondary pollen presentation, total interpetiolar stipules, and contorted aestivation [5, 6]. Fruit characters, number of seeds per carpel, and the number of ovules are of low taxonomic use because of high variations within the group [10]. Interestingly, handful of previous studies have made efforts to delimit the species and clarify the phylogenetic relationships using morphological and molecular data at different taxonomic levels [5, 6].

Previous molecular phylogenetic studies based on plastid (m*atK, ndhF, rbcL, rps16* intron*, trnS-G, trnT-F, rbcL,* and *atpB-rbcL)* and nuclear DNA data (nrITS) have shed light on the evolutionary circumstance of the Coffeeae alliance [7, 8]. In most reports, some tribes, such as Airospermeae, Alberteae, Augusteae, Bertiereae, Coffeeae, Octotropideae, and Pavetteae, have been described as monophyletic while the polyphyletic status of tribe Gardenieae remained obscured. Additionally, the clade support was generally low indicating many unknown relationships and internal resolution. Furthermore, several genera such as *Burchellia* R.Br*., Didymosalpinx* Keay, *Mantalania* Capuron ex J.-F.Lero and *Monosalpinx* N.Hallé nested within the tribe failed to appear to be related to any of the described tribes [7, 8]. In addition, Previous studies have also described multiple genera relative to the tribe Gardenieae e.g., such as *Adenorandia* Vermoesen, *Aidiopsis* Tirveng, *Alleizettella* Pit, *Fosbergia* Tirveng & Sastre, *Himalrandia* T. Yamaz, *Pseudaidia* Tirveng, *Pseudomantalania* J.-F.Leroy, and *Sulitia* Merr. Hence, urgent taxonomic reassessment via molecular phylogeny is required to understand the pattern of evolutionary relationships [5, 8]. On the other hand, in the study by Mouly et al., a revised tribal description is given for the Gardenieae by using plastid loci. The Alibertia and Sherbournia groups are recognized at the tribal level as extended Cordiereae and Sherbournieae [8]. Therefore, further studies are needed to confirm the two tribes. Overall, molecular phylogenetic studies of the Coffeeae alliance using few markers (plastid regions and nrITS) have often failed to resolve the phylogenetic relationships at tribal/generic levels and yield a well-supported tree [7, 8, 10].

In recent years, the emergence of Next Generation Sequencing (NGS) technology has improved the availability of genome data, including the plastome, to address phylogenetic relationships and understand the potential roles of the plastome structural variations (PSVs) in different plant lineages. Plastome has a quadripartite structure consisting of a large single copy (LSC) region, a small single copy (SSC) region, and a pair of inverted repeat (IR) regions [12, 13]. The PSVs, including the IR contraction and expansion, gene loss and duplication, pseudogenization, and inversions have been substantiated in many angiosperm families, e.g., Geraniaceae [14], Leguminosae [15, 16], and Rubiaceae [1, 11, 17]. A recent study has explored the PSVs within the Coffeeae alliance tribes of subfamily Ixoroideae and revealed frequent inversions, gene duplications, gene loss/pseudogenization, IR expansion/contraction, and loss of one IR in the plastomes [11]. Also, previous phylogenetic studies using few plastid loci, nuclear data and a limited number of species could not fully substantiate the tribe-level phylogenetic relationships of the Coffeeae alliance tribes [1, 11, 17]. For instance, the sister relationships of the tribes Cordierinae and Octotropideae, as well as that of the tribes Gardenieae with Pavetteae and Sherbournieae were unresolved. Thus, studying further plastomes of the representative species can provide deep insights into the PSVs alongside their phylogenetic implications and clarify the prominent phylogenetic relationships of the Coffeeae alliance.

In this study, 71 (40 newly sequenced and assembled and the remainder from the Gene bank) plastome data. We aim to explore the PSVs and reconstruct the phylogenetic tree of the Coffeeae alliance using four data matrices: coding sequences (CDs), noncoding sequences (NCDs), protein-coding genes (PCGs), and complete plastome (CP) sequences. The following key challenges were systematically explored: (1) investigation of structural variation and screening of promising marker(s) from the plastome data to provide a way forward.; (2) Identification of PSV traits and taxonomic implications; (3) Establishment of a well-supported phylogenetic framework from the partitioning schemes of the plastome data, (4) Examination of the relationships of the Coffeeae alliance tribe and comparison with previous gene fragment studies of plastid phylogenomics. The results of this study will provide insights into the plastome evolution and phylogenomics of the Coffeeae alliance tribes.

## Results

### Plastome Organization of the Coffeeae alliance tribes

**Fig. 1:**
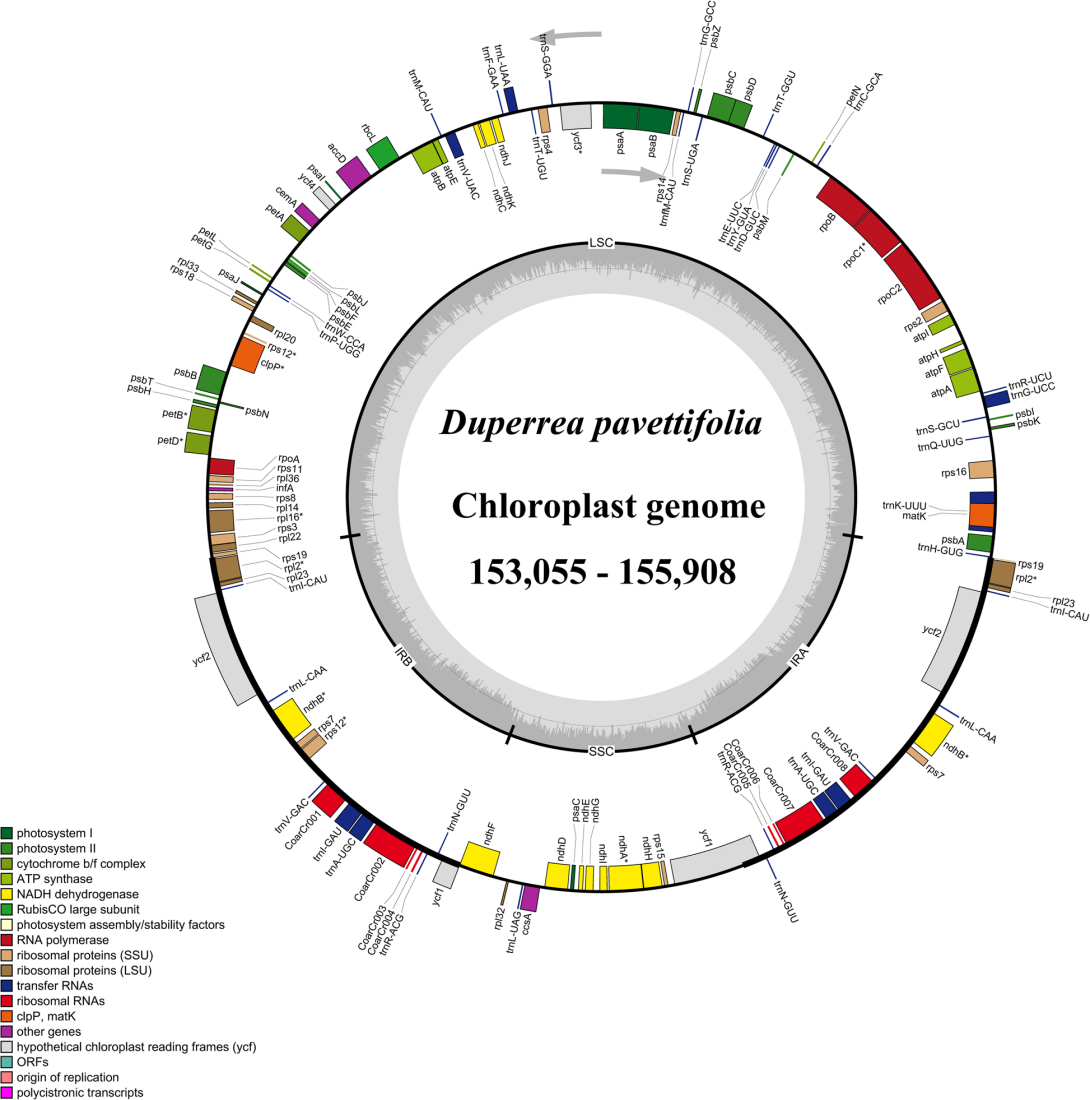
Gene map of *Duperrea pavettifolia* plastome, representative species from the Coffeeae alliance clade. Genes inside the circles are transcribed clockwise, while genes outside the circles are transcribed counter-clockwise. The light grey inner circle and the dark grey correspond to the AT and GC content, respectively. Genes belonging to different functional groups are shown with different colors

Mean coverage of the newly sequenced plastome ranged between 165.7 × (*Pavetta barbertonensis* Bremek. of Pavetteae) and 635 × (*Rubovietnamia aristata* Tirveng. of Gardenieae) (Table S1). The Plastomes of species from the Coffeeae alliance tribes exhibited a typical quadripartite structure (Fig. 1) except for *Feretia aeruginescens* Stapf of Octotropideae, in which one of the IRs is absent (Fig. S1). The plastome lengths varied from 129,434 bp in *Feretia aeruginescens* to 155,908 bp in *Rothmannia manganjae* (Hiern) Keay in Gardenieae. Likewise, there were apparent variations in the size of the plastome regions (Table S2); LSC from 83,586 bp (*Pavetta abyssinica* Frese, Pavetteae) to 85,556 bp (*Diplospora dubia* (Lindl.) Masam, Gardenieae), SSC from 15,216 bp (*Diplospora dubia*, Gardenieae) to 18,229 bp (*Fosbergia shweliensis* (J. Anthony) Tirveng. & Sastre, Gardenieae), and IR from 25,138 bp (*Bertiera breviflora* Hiern, Bertiereae) to 25,937 bp (*Feretia aeruginescens*).

Additionally, we observed a marginal difference in the GC content within the range of 36.1% (*Feretia aeruginescens*) to 37.8% (*Rubovietnamia aristata*) (Table S2). Typically, the observed plastomes contain 131 genes, 84 CDS, 37 tRNA, and 4rRNA, Exon 33, and Intron 15, but in the plastome of *Feretia aeruginescens*, 114 genes, 79 CDS, 37 tRNA, and 4 rRNA, Exon 30 and Intron 12 were detected (Table S2). Among the 11 genes with introns detected in our plastomes analyses, five had double introns (*clpP*, *ndhF*, *rpl2*, *rps12*, and *ycf3*), while six had single intron (*ndhA*, *ndhB*, *petB*, *petD*, *rpl16*, and *rpoC1*) (Table 1). In addition, the *rps12* gene trans-spliced where one intron was shared between two exons, which was duplicated in the IR region. Also, the pseudogenization of *ycf1* and *rps19* genes was observed (Fig. S1).

**Table 1:** Plastome content of Coffeeae alliance tribes

Gene group	Gene
Subunit of acetyl-CoA	*accD*
Large subunit of ribosomal proteins	*rpl2*^+,^*, rpl14, rpl16^*+,#*^, rpl20, rpl23*, rpl32, rpl33, rpl36
Small subunit of ribosomal proteins	rps2, rps3, rps4, rps7*, rps8, rps11, rps12^*++*,^*, rps14, rps15, rps16, rps18, rps19^*Ψ,#*^
Ribosomal RNA genes	rrn4.5*, rrn5, rrn16*, rrn23*
Transfer RNA genes	trnA-UGC*, trnC-GCA, trnD-GUC, trnE-UUC, trnF-GAA, trnfM-CAU, trnG-GCC, trnG-UCC, trnH-GUG, trnI-CAU*, trnI-GAU*, trnK-UUU, trnL-CAA*, trnL-UAA, trnL-UAG, trnM-CAU, trnN-GUU*, trnP-UGG, trnQ-UUG, trnR-ACG*, trnR-UCU, trnS-GCU, trnS-GGA, trnS-UGA, trnT-GGU, trnT-UGU, trnV-GAC*, trnV-UAC, trnW-CCA, trnY-GUA
DNA-dependent RNA polymerase	*rpoA, rpoB, rpoC1*^*+*^*, rpoC2*
Photosystem I	*psaA, psaB, psaC, psaI, psaJ*
Photosystem II	*petA, petB*^*+*^*, petD*^*+*^*, petG, petL, petN*
Cytochrome b/f complex	*psbA, psbB, psbC, psbD, psbE, psbF, psbH, psbI, psbJ, psbK, psbL, psbM, psbN, psbT, psbZ*
ATP synthase	*atpA, atpB, atpE, atpF, atpH, atpI*
Maturase K	*matK*
Envelope membrane proteins	*cemA*
subunit of acetyl-CoA	*accD*
RubisCo large subunit	*rbcL*
Hypothetical reading frames	*ycf1*^*Ψ,#*^, ycf2*, ycf3^*++*^*, ycf4*
Protease	*clpP*^*++*^
NADH dehydrogenase	*ndhA*^*+*^*, ndhB*^+,^*, ndhC, ndhD, ndhE, ndhF^*++*^*, ndhG, ndhH, ndhI, ndhJ, ndhK*
c-type cytochrome	*ccsA*

Note: +, single intron; ++, double introns; *, gene in the IR; Ψpseudogenes; #, duplicated

### Plastome structural variations in the Coffeeae alliance tribes

**Fig. 2:**
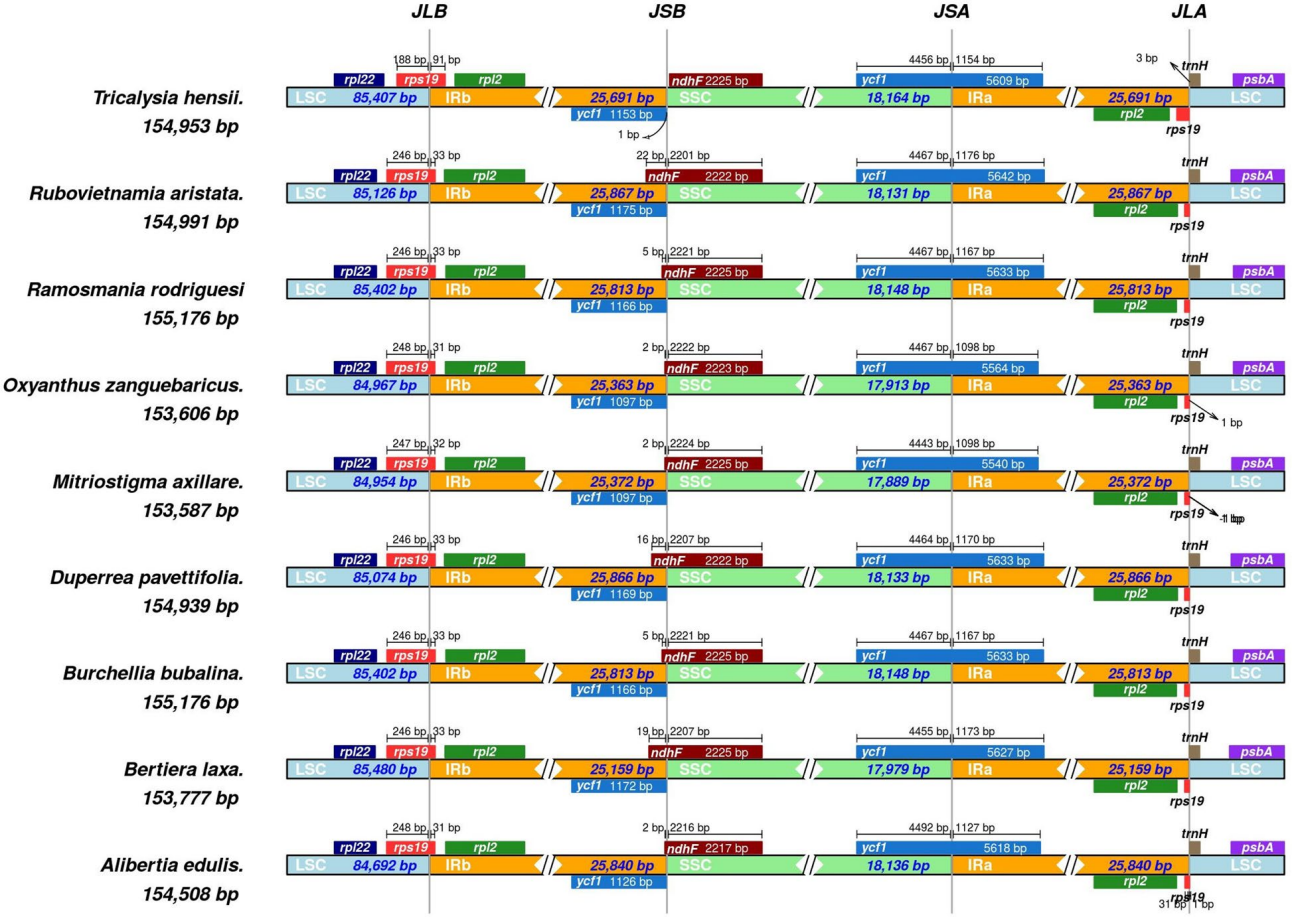
Observed negligible *ndhF* genes shift of IRB/SSC boundaries of representative chloroplast genomes of the Coffeeae alliance tribes. The IR, LSC, and IR border regions of chloroplast genomes for Coffeeae alliance tribe are indicated. Numbers below and above the gene indicate a gap between gene ends and boundary site in base lengths (bp). The genes are denoted by colored boxes

Extensive IR expansion and contraction were recorded among the Coffee alliance tribes (Fig. 2, Fig. S1). The LSC/IRB (JSB) junction, which is located within the *rps19* gene led to the gene duplication in the IRA region with lengths extending from 27 bp in *Diplospora dubia to* 95 bp in *Coffea canephora* Pierre ex A.Froehner. Also, the observed IRB gene duplication of SSC/IRA (JSA) junction largely lies within the *ycf1* gene ranging from 987 bp in *Leptactina leopoldi* Buettner to 1199 bp in *Tarenna drummondii* Bridson. Mainly, the SSC/IRB junction (JSB) is located between the duplicated *Ψycf1* (in the IR) and *ndhF* genes (in the SSC) except in few species, where the IRB is partially expanded into the *ndhF* gene in the IR (1 to 22 bp) (Fig. 2). The LSC/IRA junction (JLA) was located between duplicated IRA boundary of *Ψrps19* (in the IR) and *trnH-GUG* genes (in the LSC). However, the location of the *trnH-GUG* gene varied at the JLA border in different lengths (1 to 3 bp). No significant gene rearrangements were observed in the studied plastomes.

### Identification of sequence divergence

**Fig. 3:**
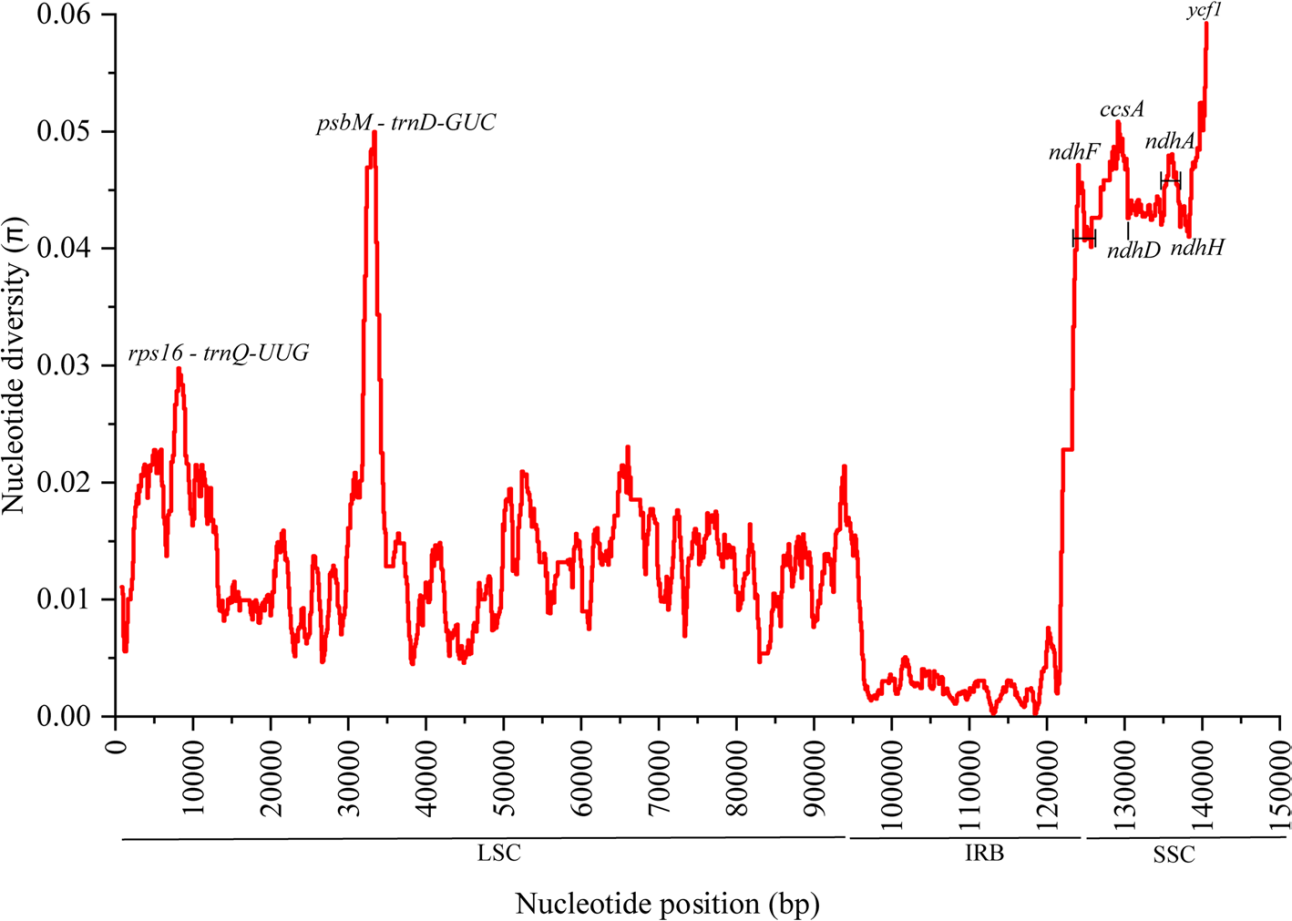
Sliding-window analysis of nucleotide variability (Pi) across the sampled 62 plastome sequences of the Coffeeae alliance including the single copy of IR

It is noteworthy that the mutation and parsimony-informative site (PIS) were different in the observed plastome. A relatively large number of variable regions (mutation hotspots) were identified, where a high rate of nucleotide substitutions may occur. These include two intragenic regions and six gene regions with an informative parsimony site between 0.02977 (*rps16 - trnQ-UUG*) and 0.05927 (*ycf1*) (Fig. 3). In addition, the informative sites in the IR region was lower than the SC regions (Fig. 3).

### Codon usage and repeat analyses in the tribes of the Coffeeae alliance

**Fig. 4:**
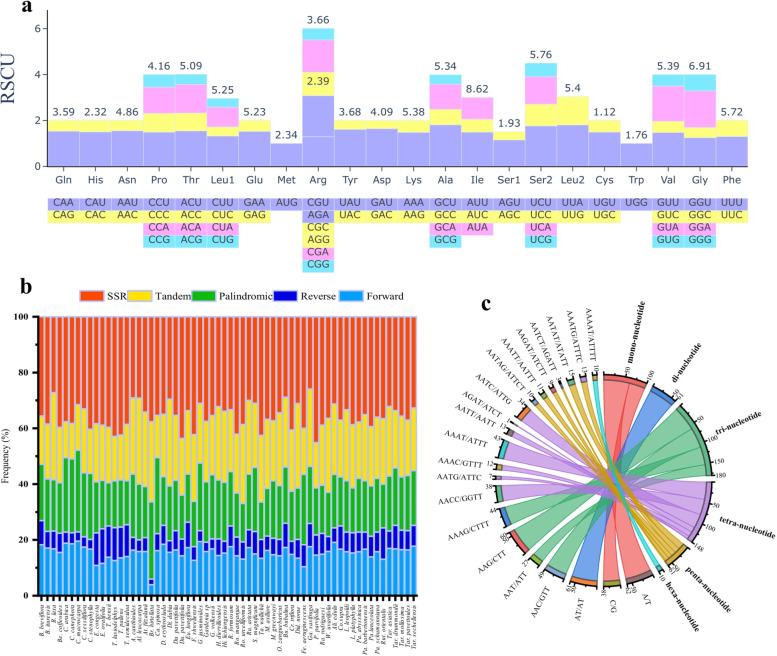
Codon and repeat analyses of the 62 sampled plastomes from the Coffeeae alliance. **a** Codon usage biases of the plastome of *Alibertia edulis*, a representative species from the Coffeeae alliance clade. The values at the top of each stack indicate the frequency of use of each amino acid, while the bars (colour-coded) represent the relative values of synonymous codon usage for each codon. **b** The percentage distribution of five types of repeats. **c** Chord diagram showing the number and type of SSRs

Codon usage and amino acid abundances in the tribes of the Coffeeae alliance were similar (Fig. 4a, Fig. S2). The total sequence of encoding protein genes ranged between 74,682 bp in *Empogona ovalifolia* (Coffeeae) to 79,249 bp in *Rothmannia manganjae* (Gardenieae). For the protein-coding genes, codons ranged from 22,573 bp in *Feretia aeruginescens* (Octotropideae) to 26,304 bp in *Atractocarpus fitzalanii* (F.Muell.) Puttock (Gardenieae) (Fig. S2). The most abundant amino acid, isoleucine Codon usage, ranged from 5.8 to 8.75% and the least abundant amino acid, cysteine Codon usage, averaged from 1.1 to 1.17% in Coffeeae alliance tribes (Fig. S2). The majority of amino acid codons have a bias, although codons AU(T)G and U(T)GG, which encode methionine (Met) and tryptophan (Trp) respectively, both show no codon preferences (RSCU = 1.00). Additionally, most types of preferred synonymous codons (RSCU > 1.00) possessed C- or U-ending codons (Fig. S2). The percentage Effective Number of Codon (Enc) and Codon Bias Index (CBI) ranged from 55.01 to 55.24% and 0.20 to 0.25%, respectively (Table S5).

We recorded five repeat types within the studied plastomes and their percentage distribution was strikingly different (Table S4). We recorded five repeat types within the studied plastomes and their percentage distribution was strikingly different Forward repeats from 4 to 20%, Reverse repeats from 2 to 12%, Palindromic repeats from 13 to 29%, Tandem repeats from 15 to 31% and SSR repeats ranging from 29 to 45% (Table S4). In addition, of the total frequency of the repeats in the sampled group, the Dispersed repeats accounted for approximately 19% (of which 19% were palindromic, 15% Forward, and 7% Reverse), Tandem repeats for 23%, and SSR for 36% (Fig. 4b).

Microsatellite analyses of the Coffeeae alliance tribes identified a total of 2588 SSRs (Table S4). Our result showed a variable distribution within the tribe ranging from 60 in *Himalrandia lichiangensis* (W.W.Sm.) Tirveng*.* (Gardenieae) to 28 in *Polyshpaeria parvifolia* (Octotropideae) (Fig. 4b). Of the six SSR motif types, penta-nucleotide (*n* = 7) was the most common while only one hexa-nucleotide was recorded. The analyses also revealed that the mononucleotide (A/T) motif was present in all the plastomes contrasting the tetra-nucleotide (AGAT/ATCT) motif that was only found in *Mitriostigma axillare* Hochst. (Fig. 4c). The A/T motifs accounted for more than 19%, and the other motif types occurred in less than 10% (Fig. 4c). In addition, mononucleotide repeats accounted for (71%) and hexanucleotides were the least common (Table S4).

### Phylogenetic relationships of the Coffeeae alliance tribes

**Fig. 5:**
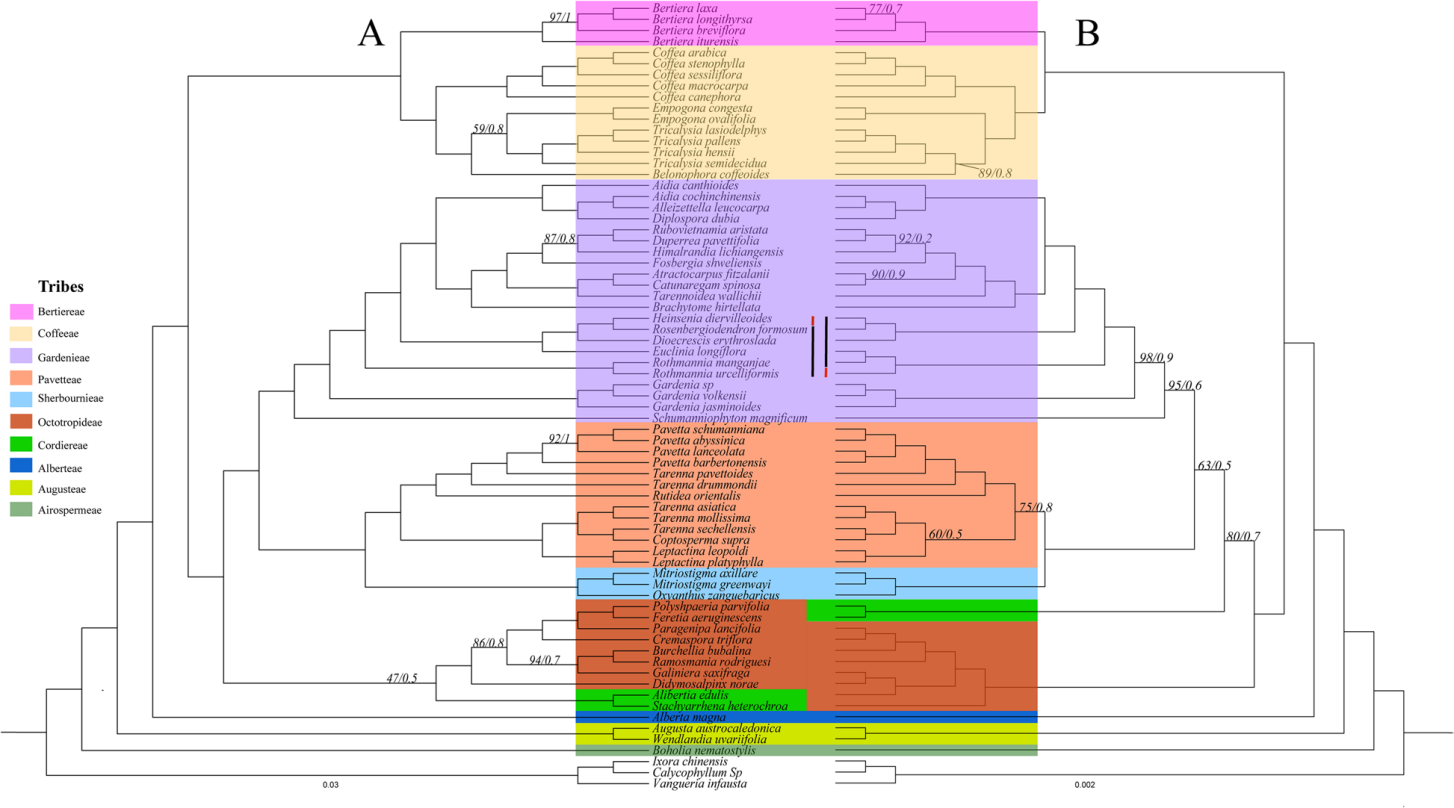
The reconstructed phylogenetic tree of the Coffeeae alliance tribes using the plastome data. **a** CP and NCDS. **b** CDS and PG. Colored boxes indicate tribe classification. The thick black line represents the topological differences within the tribe Gardenieae. Support values (BS/PP) either < 100 or < 1 are shown above the branches

The phylogenies inferred from four data matrices using two tree methods (ML and BI) yielded nearly similar topologies. Our plastid phylogenomic analyses supported the monophyly relationships of the Coffeeae alliance tribes and resolved relationships of all the deep nodes, with the strong support values (ML =100%, BI =1.0, Fig. 5). However, few subclades, namely the tribes Cordiereae + Octotropideae (ML =47%, BI = 0.5, Fig. 5a) and *Belonophora* — *Empogona* of the tribe Coffeeae (ML =59%, BI =0.8, Fig. 5a) are weakly supported. Also, the analyses presented well-supported sister relationships of the tribe Airospermaeae to the remaining Coffeeae alliance tribes. The tribe Augusteae formed a sister relationship with the tribe Albertieae, as a sister tribe to the lineages consisting of tribes Octotropideae and Cordiereae.

The tribe Cordiereae has different phylogenetic positions within the entire clade of the Coffeeae alliance from the data matrices, especially the data from the CDs and NCDs compared to other data matrices (Fig. 5). Within the Coffeeae alliance clade, the phylogenetic position of the tribe Octotropideae in the CP and NCDs trees with weak support values was attributed to a sister relationship with the Corderieae (Fig. 5a), whereas the monophyletic relationship of this tribe is well-supported in the trees reconstructed from the PG and CDs (Fig. 5b). The analyses supported the sister relationships of the tribes Sherbournieae-Pavetteae clade, sister to the monophyletic tribe Gardenieae with high support values (ML = 100%/BI = 1 Fig. 5b). In addition, our analyses strongly supported the relationships of the tribes Coffeeae and Berticereae. Our data further revealed the topological differences at genera and species levels, which can easily be observed from *Heinsenia diervilleoides* K.Schum. located in different positions according to the molecular data matrices (Fig. 5).

## Discussion

### Evolutionary pattern of PSVs in the Coffeeae alliance tribes

Plastomes structural variations in the subfamily Ixoroideae have previously been documented [11]. Moreover our comprehensive sampling of the plastomes from the Coffeeae alliance tribe further enhances the understanding of its plastome evolutionary history. From our analyses, no gene loss was detected hence; there are marginal differences between the sizes of studied plastomes in the Coffeeae alliance tribe. Contrary to our results, loss of the *trnH-GUG* gene has been reported in *Tarenna grevei* (Drake) Homolle of the subfamily Ixoroideae [11]. Previous reports have revealed the potential implications of gene loss (single or multiple) in the plastome size variations. It has been suggested in previous studies that gene losses in the plastomes are attributed to the rate of sequence evolution, gene transfer into the nucleus, or substitution of a plastid gene product with a nuclear-encoded protein [18, 19].

In our study, we suspected two pseudogenes (*rps19* and *ycf1*), based on the presence of premature stop codons and their short length. This is synapomorphy to the Coffeeae alliance tribes (Fig. S1). The observation is consistent with the previous studies where the pseudogenization of *rps19* and *ycf1* genes have been detected in many angiosperms [11, 20–22]. Pseudogenization is an evolutionary process in plastid genomes, which might occur during gene transfer to the nucleus, substitution, and nuclear-encoded protein for a plastid gene product source of DNA barcodes [23–25]. Our observation on the trans-spliced *rps12* gene (exon 1 and exons 2) is congruent with the previous report [26]. During genetic evolution events, it has been proposed that exons, which are the gene fragments encoding for protein structural domains are impacted by reorganization into other genes [27]. Additionally, as a result of RNA splicing for *rps12* exon 1 with transcripts from other genes, and polypeptide variations in the plastome have been reported [27]. Therefore, it could be suggested that similar events can explain the trans-splicing events of *rps12* gene in the plastomes members of the Coffeeae alliance tribes.

The observed IR loss and occurrences of multiple IR contraction/expansion in the studied plastomes agrees with a previous study of the subfamily Ixoroideae [11]. In our investigation, IR contraction/expansion varies at the boundaries, and this character is synapomorphy of the Coffeeae alliance (Fig. S1). For instance, the loss of one IRs or expansion in *Feretia aeruginescens species* resulted in a larger IR size. The evolution of plant lineages is a dynamic process IR contraction/expansion at borders is the lineage with plastome genomes and plays an essential role in revealing the evolution events [28–30]. The terminal IR gene adjacent to the SSC region is highly conserved, however, there were variations at the IR/LSC boundary [31–33]. The rate of gene conversions during cell division/evolution and high concentration of short repeats (AT-rich) are the widely accepted explanation of the IR boundary shifts among several angiosperm lineages [34, 35]. In our study, the *ndhF* gene independently shifts at IRB/SSC border of the Coffeeae alliance plastomes (Fig. 2). Other previous studies have also revealed that unique boundary shifts of the IR and SSC regions are correlated with the transformations of *ndhF* and *ycf1* genes [35–37]. The IR boundary shifts are widely accepted mechanisms for the plastomes rearrangements, which contributes to the structural diversification in plastomes [35].

Plastome sequencing has been used to infer relationships at all taxonomic levels, for example, to reveal relationships at the lowest level in land plants [36]. Two plastid protein coding genes, *rbcL* and *matK*, and the *psbA*-*trnH* intergenic spacer, have been recommended as universal plastid DNA barcodes for land plants [37]. Seven promising plastid DNA barcode (*matK, accD, rps19, ndhF, ndhD, ccsA* and *ycf1*) have been consistently consider the most variable regions for different plant [38–41]. It was important to determine whether these loci could be used as potential molecular markers to establish relationships between species and populations of Coffeeae allinese tribes. Our results showed that the IRs had negligible sequence divergence compared to the SC regions, which also occurred in higher plants and possibly due to copy correction between IR sequences by gene conversion [42] .

Codon usage has shown that plastomes evolve due to a high frequency of privileged mutations in the coding sequences in the evolution of species [43]. The RSCU values are affected by the mutation factor that affects the codon usage pattern. Thus, Relative synonymous codon usage with value < 1 indicates that codons are used less frequently, > 1 represents codons that are used more frequently, and value =1 indicates no bias [44]. We found identical trend in the pattern in which the amino acids were encoded for the 62 species (Fig. S2). The most abundant amino acid, isoleucine, and the trend are consistent with other angiosperm plastomes, where leucine and isoleucine were the most abundant codons [43, 45]. The Effective Number of Codons (ENc), which ranges from 20 (one codon per amino acid) to 61 (equal use of synonymous codons) [46], and the Codon Bias Index (CBI), which ranges from 0, no bias, to 1, equal use of all synonymous codons [47]. In our study, the values for ENc (55 to 55.2) and CBI (0.20 to 0.251) differed insignificantly among species of Coffeeae alliance consistence with other report [48, 49].

The high degree of polymorphism and genome-wide distribution, such as significant variation in motifs and with several repeats, can be studied using SSRs analyses. Especially in determining genetic diversity and population genetic issues such as gene flow, ancestry, and population structure [50]. From the comparative analysis of five repeats in the Coffeeae alliance tribe, SSRs are predominantly mononucleotide (A/T) motifs. A similar trend of SSR distribution was also confirmed in the chloroplast genome of other angiosperms [43]. Other downstream analyses, repeats in a system with various rearrangements, deletions, additions, and large inversions within the cp genome are well known [51]. The percentage distributions of Tandem repeats were abundant from our analyses (Fig. 4b). Several studies have shown that a high number of repeats positively correlates with the PSV or plastome size of angiosperms. Noteworthy, a high number of Tandem repeats are important for structural reorganization of the plastome and a wide range of IR expansions/contractions [52].

### Phylogenetic relationship of Coffeeae alliance tribes

Our results confirm that ML and BI analyses with multiple genes partitioned models (CDs, NCDs, PG, and CP) could resolve the phylogeny relationships of the Coffeeae alliance tribes (Fig. 5). The most recent phylogenetic studies have reported improved phylogenetic resolutions of the Coffeeae alliance, but with only a limited number of representatives [1, 11, 17]. Again, our study punctuated the importance high taxonomic sampling for phylogenetic relationships as we included more than half of the total genus to achieve deep phylogenetic resolution of the Coffeeae alliance tribes. The phylogenetic relationships among key lineages, genera, and tribes were resolved with strong support values (Fig. 5) and are consistent with previous studies [8, 11, 17, 53]. Our multi-locus analyses showed that the monophyletic Airospermeae is the first diverged lineage followed by Augusteae, Alberteae, Cordiereae, Octotropideae, Sherbournieae, Pavetteae, Gardenieae, Coffeeae, and Bertiereae (Fig. 5) coinciding with recent findings [1]. Our phylogenetic analyses for all the datasets agree with previous analysis based using CDS, and combined CDS + non CDS on the phylogenetic position of the tribe Augusteae (ML = 100%/BI = 1) with full support non CDS [1].

Despite the conflicting phylogenetic positions of the tribes Cordiereae and Octotropideae (Fig. 5), the sister relationship of both tribes is largely consistent with the recent phylogenetic analyses inferred based on the plastome data [1]. Herein, we revealed the tribes Pavetteae, Sherbournieae, and Gardenieae have monophyletic relationships, including most of the genera within the tribes with the highest support values (ML = 100%/BI = 1). In previous studies, analyses of CDS, combined with CDS + non CDS resulted in different topologies for the tribe Gardenieae, Sherbournieae and Pavetteae. In addition, the use of plastid data in the tribe Gardenieae poorly supported sister relationship with the tribes Sherbournieae and Pavetteae. However, the tribe Pavetteae showed an unsupported sister relationship to Sherbournieae, which contrasts with the tribes Sherbournieae and Gardenieae, that revealed polyphyletic based on plastid and nuclear data [1, 11, 17]. Our phylogenomic analyses have shown that the relationship of the tribe Pavetteae and tribe Sherbournieae is strongly supported (Fig. 5). Besides, this study strongly supports the sister relationship between the tribes Coffeeae and Berticereae, which agrees with the other studies [1, 11, 17, 54].

Previous studies using the morphological characters of the genera (*Alleizettella* Pit, *Fosbergia* Tirveng & Sastre and *Himalrandia* T. Yamaz) have a limited role in the classification of the tribe Gardenieae [8, 55]. Our phylogenomic analyses reveal a deeper phylogenetic resolution of the Coffeeae alliance tribe. We confirmed the phylogenetic placement of *Fosbergia shweliensis*, *Himalrandia* lichiangensis, and *Duperrea pavettifolia* (Kurz) Pit. Within the monophyletic tribe Gardenieae with strong support (ML = 87%/BI = 0.8; Fig. 5). Interestingly, the presence of morphological features such as terminal inflorescences and absent of Raphides seems to further reinforce the relationships of *Duperrea pavettifolia* and tribe Gardenieae, [8, 55]. In addition, previous phylogenetic studies of the Coffeeae alliance exploiting several plastid and nuclear markers have failed to resolve the relationships between several genera (e.g., *Burchellia, Didymosalpinx,* and *Galiniera Delile*) and their phylogenetic status with members of the tribes Sherbournieae and Cordiereae [5, 8]. Our finding moderately supported the close relationships of these genera with the tribe Octotropideae whereas the basal lineage, *Didymosalpinx norae* (Swynn.) Keay formed a sister relationship with the remaining genera of the tribes (Fig. 5). Thus, our study provides an improved understanding of the phylogenetic relationships in the Coffeeae alliance. Also, the study shows the potential of plastomes in resolving intertribal and intergeneric relationships within the Coffeeae alliance tribes. However, further phylogenetic studies of the tribes Cordiereae and Sherbournieae, perhaps integrating additional molecular data and morphological traits would fully clarify the evolutionary relationships of the Coffeeae alliance clade.

## Conclusion

The study detected several PSVs which have occurred independently across the lineages but without clear taxonomic and phylogenetic implications. Rather, they played an important role in the structural restructuring of the plastome in the Coffeeae alliance tribe. Furthermore, we screened promising molecular markers in both the intragenic and coding regions which might be suitable for future phylogenetic studies for the Coffeeae alliance clade. We utilized the plastome data to present a well-resolved phylogenetic tree at the tribal and generic levels for the Coffeeae alliance tribes. Thus, the results obtained provide significant insight into the PSVs and phylogenetic relationships of the Coffeeae alliance and the Rubiaceae family and open a way for robust phylogenetic studies in future.

## Materials and methods

### Taxon sampling, DNA extraction, and genome sequencing

A total of 71 plastomes were used for this study, including 68 sequences from the alliance group of Coffeeae alliances (representing 44 genera), and three outgroups. Of these, 31 plastomes were obtained from GenBank (www.ncbi.nlm.nih.gov/genbank) and the remaining 40 were newly generated for this study (Table S1). For the newly sequenced taxa, total genomic DNA was extracted from silica-gel dried leaves using plant genomic DNA kit based on the manufacturer’s instructions. The genome skimming technique was used to obtain the plastome data using fragmented gDNA data; the library size 350 bp inserts were selected. Sequencing with 2 × 150-bp paired-end (PE) read was performed on the Illumina Hiseq 2500 platform at the Novogene Company (Beijing, China).

### Plastomes assembly and annotation

The clean-up Raw and quality checks were performed using the NGS QC Tool Kit [56]. De novo assembly of the cleaned reads was performed in GetOrganelle with K-mer values of 21, 45, 65, 85 and 105 GetOrganelle, using the plastome of *Coffea arabica* L. (NC008535) as reference [57]. Subsequently, the assembled reads were visualized and filtered in Bandage v.0.80 [58] to generate a complete plastome. For incomplete plastomes, we filled the gaps between contigs with consensus sequences of raw reads mapped initially to the reference plastome to obtain the complete plastome. The number of mapped paired-end (PE) reads and the depth of coverage was determined by mapping the paired reads against the plastome using Bowtie2 [59] The locations of single copies (SC) and IR boundaries in the resequenced plastomes were determined using in Geneious v. 8.1.4 [60]. The find repeat function in Geneious was used to flank the IR regions. Then, the paired reads were reassigned to the assembled plastomes to validate the SC /IR regions with Bowtie2 [59]. Finally, we visualized the read stacks of the reassembled plastomes and compared the labeled SC/IR boundaries in Geneious. The new plastomes were annotated using the Dual Organellar Genome Annotator web interface (DOGMA) [61]. We manually checked the consistency of start/stop codons and intron/exon boundaries in Geneious. The Find ORFs function in Geneious was used to confirm protein-coding gene (PCG) annotations, while the tRNAscan-SE web service was used to determine tRNA genes [62]. The OrganellarGenomeDRAW web server was used to produce circular maps for the newly sequenced plastomes [63]. We also deposited the 40 newly assembled plastome sequences from the Coffeeae alliance and outgroup in GenBank (Table S1).

### Plastome structural analyses

To investigate the patterns of plastome genomic evolution of the Coffeeae alliance clade, 62 annotated plastomes were compared and the length (bp) of the plastome and its elements (LSC, SSC, and IR), gene contents (number of genes), and GC contents were summarized (Table S2, Fig. S1). Comparative analyses of the SC/IR boundary shifts at four junctions (JLB*-*LSC/IRB, JSB*-*IRB/SSC, JSA*-*SSC/IRA, and JLA*-*IRA/LSC) of the sampled plastomes was conducted in Geneious and plotted in IRscope [60, 64]. We investigated the potential rearrangements in gene order (e.g., inversion, IR boundary shifts) for all the sampled species of the Coffeeae alliance using *Coffea arabica* as the reference. This analysis was performed in Mauve v.2.3.1 [65] using default settings: automatically calculate the seed weight (15) and Locally Collinear Blocks (LCBs) with the minimum LCB score of 30,000 [65].

### Sequence divergence, codon usage and repeat analyses

To assess sequence divergence and examine highly variable regions in the plastome, nucleotide diversity (π) was calculated by sliding window analysis using DnaSP v6 [66] with a window size of 1000 bp and a step size of 100 bp. The level of codon usage bias was determined by analysing the Relative Synonymous Codon Usage RSCU [67];, Effective Number of codon ENc [46]; and the Codon Biased Index CBI [47]; for all PCGs, in DnaSP 6.10 [66]. The average RSCU and amino acid frequency values for each species were plotted using R-script (Fig. S2) [68].

We searched for three repeat types, compositions, and distributions within the sampled 62 plastomes from the Coffeeae alliance tribes. Tandem repeat distribution were identified using Tandem Repeats Finder v4.09 [69] with the following settings: alignment parameters match (2), mismatch (7), indels (7), minimum alignment score (80), and maximum period size (500). Disperse repeats (forward, reverse, complementary, and palindromic) were also detected via REPuter [70] with a minimum repeat size of 30 bp and a Hamming distance of 1 (Table S3). The simple sequence repeat (SSRs or microsatellites) motifs in the plastomes were searched using the Perl script using MISA v2.0 [71]. W set the minimum numbers (thresholds) of the SSRs for mono-, di-, tri-, tetra-, penta-, and hexanucleotides, to 10, 6, 5, 5, 5, and 5, respectively (Table S3).

### Phylogenetic analyses

Phylogenetic analyses included a total of 71 plastomes data (68 ingroups and 3 outgroups). We performed individual gene alignment to overcome poor alignment challenges from the whole genome and generate different data matrices for the phylogenetic analyses. To achieve these, we extracted coding (CDs) and noncoding (NCDs) regions in Phylosuit v1.1.13 [68], followed by an individual gene alignments using MAFFT v7.450 [72] with LINSI algorithm and concatenation in Geneious [60]. To minimize the systematic error, we excluded NCDs loci with less than 80% taxon occupancy and alignment lengths less than 100 bp. The data matrices were generated to reconstruct the phylogenetic relationships of the Coffeeae alliances: CDs (84 genes), NCDs (122 loci), PG (78 genes), and CP (concatenated CDs and NCDs for all species). Substitution models of the four matrices were determined using Partition Finder v2.1.1 [73], and the best evolutionary models were selected using the Akaike Information Criterion (AIC) (Table S2). The phylogenetic relationships were reconstructed using Maximum Likelihood (ML) and Bayesian Inference (BI) tree methods. The ML analyses were performed in RAxML-HPC2 v.8.1 on XSEDE [74] with the GTRCAT substitution model and 1000 Bootstrap replicates. The BI analyses were inferred using MrBayes v.3.2.6 [75]. We estimating the Bayesian posterior probability (PP) with two independent Markov Chain Monte Carlo (MCMC) runs, via a single cold chain and three hot chains for 10,000,000 generations, while sampling the tree at every 1000 generations. We assessed the MCMC convergence of each parameter for every run based on sufficient effective sample size (ESS ≥200). The first 20% runs were discarded as burn*-*in using TRACER v.1.7 [76]. The resulting trees (ML and BI) are visualized and edited in Fig tree v.1. 3.1 [77].

## Supplementary Information

**Additional file 1.** The Mauve alignment of the 62 coffeeae alliance plastomes. The colliner blocks rearrangement of the accessible gene at junction of inverted repeat and small single copy. White colored blocks: protein coding gene, Black colored blocks: tRNA genes, Red coloured blocks: Rrna. For interpretation of references to the colors, see the web version of the article.

**Additional file 2.** The codon usage bias in all the protein-coding genes of the plastome of the 62 Coffeeae allinces tribes.

**Additional file 3: Table S1.** GenBank accession numbers and coverage of the newly sequenced species from the Coffeeae alliance studied.

**Additional file 4: Table S2.** Summary of plastomes for 63 species Coffeeae alliance tribes.

**Additional file 5: Table S3.** Summary of partitioning scheme for the ML tree and BI analysis.

**Additional file 6: Table S4.** Distributions of SSR in the Coffeeae alliance tribes.

**Additional file 7: Table S5.** Details of Relative Synonymous Codon Usage in plastome of Coffeeae alliance tribes.

## Data Availability

All sequences used in for this study can be found in GenBank; the list of accession can be found in Supplementary Table S1.
