# Supplementary material for: Phylogenomic and comparative analyses of Coffeeae alliance (Rubiaceae): deep insights into phylogenetic relationships and plastome evolution
Source: BMC Plant Biol. 2022 Feb 26;22:88. doi: 10.1186/s12870-022-03480-5 (PMC8881883; doi:10.1186/s12870-022-03480-5)
Supplement: Supplementary file 1 — Additional file 1. The Mauve alignment of the 62 coffeeae alliance plastomes. The colliner blocks rearrangement of the accessible gene at junction of inverted repeat and small single copy. White colored blocks: protein coding gene, Black colored blocks: tRNA genes, Red coloured blocks: Rrna. For interpretation of references to the colors, see the web version of the article. [file 12870_2022_3480_MOESM1_ESM.pdf]

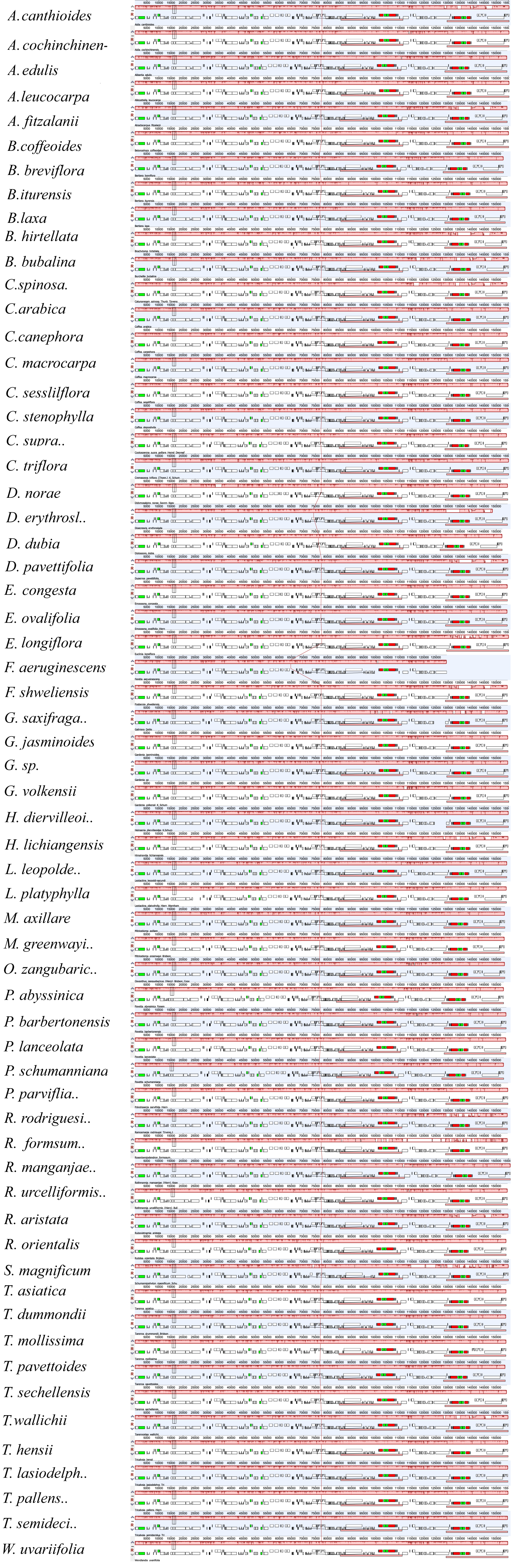


**Fig.S1.** The MAUVE alignment of the 62 coffeeae alliance plastomes. The colliner blocks rearrangement of the accessible gene at junction of inverted repeat and small single copy .White colored blocks:protein coding gene,Black colored blocks:tRNA genes,Red colored blocks : rRNA.For interpretation of references to the colors ,see the web version of the article.
