# Supplementary material for: Phylogenomic and comparative analyses of Coffeeae alliance (Rubiaceae): deep insights into phylogenetic relationships and plastome evolution
Source: BMC Plant Biol. 2022 Feb 26;22:88. doi: 10.1186/s12870-022-03480-5 (PMC8881883; doi:10.1186/s12870-022-03480-5)
Supplement: Supplementary file 3 — Additional file 3: Table S1. GenBank accession numbers and coverage of the newly sequenced species from the Coffeeae alliance studied. [file 12870_2022_3480_MOESM3_ESM.docx]

Table S1. GenBank accession numbers and coverage of the newly sequenced species from the Coffeeae alliance studied.

| Tribes | Species | Coverage | Accession Number |
| --- | --- | --- | --- |
| Coffeeae | *Empogona ovalifolia* | 336.1 X | MZ557578 |
| Coffeeae | *Tricalysia semidecidua ** | NA | MN850666.1 |
| Coffeeae | *Tricalysia lasiodelphys** | NA | MN850663.1 |
| Coffeeae | *Tricalysia hensii ** | NA | MN850665.1 |
| Coffeeae | *Tricalysia congesta ** | NA | MN850664.1 |
| Coffeeae | *Tricalysia pallens* | 456.8 X | MZ557586 |
| Coffeeae | *Coffea arabica******** | NA | NC_008535.1 |
| Coffeeae | *Coffea canephora******** | NA | NC_030053.1 |
| Coffeeae | *Coffea macrocarpa******** | NA | MK577909.1 |
| Coffeeae | *Coffea sessiliflora******** | NA | MN851272.1 |
| Coffeeae | *Coffea stenophylla******** | NA | MK577912.1 |
| Coffeeae | *Belonophora coffeoides** | NA | MN850667.1 |
| Bertiereae | *Bertiera longithyrsa******** | NA | KY348833 |
| Bertiereae | *Bertiera breviflora** | NA | MN850670.1 |
| Bertiereae | *Bertiera iturensis** | NA | MN850668.1 |
| Bertiereae | *Bertiera laxa ** | NA | MN850669.1 |
| Gardenieae | *Aidia canthioides* | *1.34 X* | MZ557597 |
| Gardenieae | *Aidia cochinchinensis* | *550.0 X* | MZ557582 |
| Gardenieae | *Alleizettella leucocarpa* | *379.4 X* | MZ557608 |
| Gardenieae | *Diplospora dubia* | *303 X* | MZ557590 |
| Gardenieae | *Rubovietnamia aristata* | *635 X* | MZ557575 |
| Gardenieae | *Duperrea pavettifolia* | *479.5 X* | MZ557574 |
| Gardenieae | *Himalrandia lichiangensis* | *371 X* | MZ557583 |
| Gardenieae | *Catunaregam spinosa* | *447.6 X* | MZ557607 |
| Gardenieae | *Tarennoidea wallichii* | *452 .1 X* | MZ557599 |
| Gardenieae | *Brachytome hirtellata* | *412.0 X* | MZ557605 |
| Gardenieae | *Heinsenia diervilleoides* | *271.8 X* | MZ557592 |
| Gardenieae | *Rosenbergiodendron formosum* | *307.7X* | MZ557602 |
| Gardenieae | *Dioecrescis erythroslada* | *349.5 X* | MZ557598 |
| Gardenieae | *Rothmannia urcelliformis* | *357.6 X* | MZ557580 |
| Gardenieae | *Rothmannia manganjae* | *502.7X* | MZ557606 |
| Gardenieae | *Gardenia volkensii* | *467.9 X* | MZ557591 |
| Gardenieae | *Schumanniophyton magnificum* | *505.2 X* | MZ557601 |
| Gardenieae | *Fosbergia shweliensis******** | NA | NC_050962.1 |
| Gardenieae | *Gardenia jasminoides******** | NA | MW160432.1 |
| Gardenieae | *Atractocarpus fitzalanii** | NA | MN850658.1 |
| Gardenieae | *Euclinia longiflora** | NA | MN850671.1 |
| Gardenieae | *Gardenia sp** | NA | MN850672.1 |
| Pavetteae | *Pavetta abyssinica* | *369.8 X* | MZ557588 |
| Pavetteae | *Pavetta lanceolata* | *505.3 X* | MZ557581 |
| Pavetteae | *Pavetta barbertonensis* | *165.7 X* | MZ557600 |
| Pavetteae | *Tarenna pavettoides* | *356.8 X* | MZ557573 |
| Pavetteae | *Tarenna drummondii* | *446.5 X* | MZ557585 |
| Pavetteae | *Rutidea orientalis* | *509.0 X* | MZ557584 |
| Pavetteae | *Tarenna asiatica* | *443.5 X* | MZ557577 |
| Pavetteae | *Tarenna mollissima* | *390.2 X* | MZ557603 |
| Pavetteae | *Coptosperma supra* | *523.3 X* | MZ557571 |
| Pavetteae | *Tarenna sechellensis* | *396.8 X* | MZ557593 |
| Pavetteae | *Leptactina platyphylla* | *459.6 X* | MZ557595 |
| Pavetteae | *Pavetta schumanniana******** | *NA* | MN851271 |
| Pavetteae | *Leptactina leopoldi-secundi ** | *NA* | MN850673.1 |
| Sherbournieae | *Mitriostigma greenwayi* | *513.1 X* | MZ557579 |
| Sherbournieae | *Mitriostigma axillare ** | *NA* | MN850674.1 |
| Sherbournieae | *Oxyanthus zanguebaricus* | *441.0 X* | MZ557587 |
| Octotropideae | *Ramosmania rodriguesi* | *515.6 X* | MZ557570 |
| Octotropideae | *Burchellia bubalina* | *402.8 X* | MZ557596 |
| Octotropideae | *Galiniera saxifraga* | *533.0 X* | MZ557572 |
| Octotropideae | *Polyshpaeria parvifolia* | *509.0 X* | MZ557604 |
| Octotropideae | *Cremaspora triflora* | *446.0 X* | MZ557594 |
| Octotropideae | *Didymosalpinx norae* | *529.1 X* | MZ557576 |
| Octotropideae | *Paragenipa lancifolia******** | NA | KY348838 |
| Octotropideae | *Feretia aeruginescens******** | NA | MN851268.1 |
| Octotropideae | *Alibertia edulis** | NA | MN850659.1 |
| Octotropideae | *Paragenipa lancifolia******** | NA | KY348838 |
| Augusteae | *Wendlandia uvariifolia* | *182.6 X* | MZ557569 |
| Augusteae | *Augusta austrocaledonica******** | NA | KY492076 |
| Condamineae | *Calycophyllum Sp* | 261.1 X | MZ557589 |
| Alberteae | *Alberta magna** | NA | KY348839 |
| Ixoreae | *Ixora chinensis** | NA | MZ221832 |
| Airospermeae | *Boholia nematostylis Merr. ** | NA | KY348840 |
| Vanguerieae | *Vangueria infausta** | NA | MN851269 |

Note:* - downloaded plastomes
