# Supplementary material for: Phylogenomic and comparative analyses of Coffeeae alliance (Rubiaceae): deep insights into phylogenetic relationships and plastome evolution
Source: BMC Plant Biol. 2022 Feb 26;22:88. doi: 10.1186/s12870-022-03480-5 (PMC8881883; doi:10.1186/s12870-022-03480-5)
Supplement: Supplementary file 4 — Additional file 4: Table S2. Summary of plastomes for 63 species Coffeeae alliance tribes. [file 12870_2022_3480_MOESM4_ESM.docx]

Table S2: Summary of plastomes for 63 species Coffeeae alliance tribes.

| Tribe | Species | Length (bp) | | | | GC (%) | Number of genes | | | | | |
| --- | --- | --- | --- | --- | --- | --- | --- | --- | --- | --- | --- | --- |
|  |  |  |  |  |  |  | Total | CDs | tRNA | rRNA | Exon | Intron |
|  |  | Plastome | LSC | IR | SSC |  |  |  |  |  |  |  |
| *Bertiereae* | *Bertiera breviflora* | 153055 | 85196 | 25138 | 17583 | 37.5 | 131 | 84 | 37 | 8 | 36 | 15 |
| *Bertiereae* | *Bertiera laxa* | 154675 | 85481 | 25159 | 17979 | 37.5 | 131 | 84 | 37 | 8 | 36 | 15 |
| *Bertiereae* | *Bertiera iturensis* | 153777 | 85399 | 25553 | 18170 | 37.5 | 131 | 84 | 37 | 8 | 36 | 15 |
| *Coffeeae* | *Coffea arabica* | 155189 | 85167 | 25943 | 18137 | 37.5 | 131 | 84 | 37 | 8 | 36 | 15 |
| *Coffeeae* | *Coffea stenophylla* | 155124 | 85226 | 25890 | 18113 | 37.4 | 131 | 84 | 37 | 8 | 36 | 15 |
| *Coffeeae* | *Coffea canephora* | 154751 | 84848 | 259009 | 18086 | 37.5 | 131 | 84 | 37 | 8 | 36 | 15 |
| *Coffeeae* | *Coffea macrocarpa* | 155186 | 85272 | 25883 | 18149 | 37.5 | 131 | 84 | 37 | 8 | 36 | 15 |
| *Coffeeae* | *Coffea sessiliflora* | 155010 | 85101 | 25901 | 18106 | 37.5 | 131 | 84 | 37 | 8 | 36 | 15 |
| Coffeeae | *Empogona congesta* | 154671 | 85106 | 25693 | 18179 | 37.5 | 131 | 84 | 37 | 8 | 36 | 15 |
| Coffeeae | *Empogona ovalifolia* | 154628 | 85074 | 25714 | 18127 | 37.5 | 131 | 84 | 37 | 8 | 36 | 15 |
| Coffeeae | *Tricalysia lasiodelphys* | 154898 | 85403 | 25680 | 18136 | 37.5 | 131 | 84 | 37 | 8 | 36 | 15 |
| Coffeeae | *Tricalysia pallens* | 155001 | 84439 | 25694 | 18175 | 37.5 | 131 | 84 | 37 | 8 | 36 | 15 |
| Coffeeae | *Tricalysia hensii* | 154953 | 85403 | 25691 | 18164 | 37.5 | 131 | 84 | 37 | 8 | 36 | 15 |
| Coffeeae | *Tricalysia semidecidua* | 154816 | 85337 | 25657 | 18164 | 37.5 | 131 | 84 | 37 | 8 | 36 | 15 |
| Coffeeae | *Belonophora coffeoides* | 155190 | 85460 | 25800 | 18131 | 37.4 | 131 | 84 | 37 | 8 | 36 | 15 |
| Gardenieae | *Aidia canthioides* | 155405 | 85487 | 25865 | 18189 | 37.5 | 131 | 84 | 37 | 8 | 36 | 15 |
| Gardenieae | *Aidia cochinchinensis* | 155389 | 85550 | 25865 | 18109 | 37.5 | 131 | 84 | 37 | 8 | 36 | 15 |
| Gardenieae | *Alleizettella leucocarpa* | 155389 | 85551 | 25865 | 18109 | 37.5 | 131 | 84 | 37 | 8 | 36 | 15 |
| Gardenieae | *Diplospora dubia* | 152495 | 85556 | 25862 | 15216 | 37.6 | 131 | 84 | 37 | 8 | 36 | 15 |
| Gardenieae | *Rubovietnamia aristata* | 154991 | 85127 | 25867 | 18130 | 37.7 | 131 | 84 | 37 | 8 | 36 | 15 |
| Gardenieae | *Duperrea pavettifolia* | 154939 | 85081 | 25863 | 18133 | 37.7 | 131 | 84 | 37 | 8 | 36 | 15 |
| Gardenieae | *Himalrandia lichiangensis* | 154972 | 85065 | 25845 | 18217 | 37.5 | 131 | 84 | 37 | 8 | 36 | 15 |
| Gardenieae | *Fosbergia shweliensis* | 154720 | 84751 | 25870 | 18229 | 37.6 | 131 | 84 | 37 | 8 | 36 | 15 |
| Gardenieae | *Catunaregam spinosa* | 154634 | 85478 | 25865 | 18189 | 37.7 | 131 | 84 | 37 | 8 | 36 | 15 |
| Gardenieae | *Atractocarpus fitzalanii* | 154627 | 84991 | 25853 | 17930 | 37.7 | 131 | 84 | 37 | 8 | 36 | 15 |
| Gardenieae | *Tarennoidea wallichii* | 155018 | 85067 | 25864 | 18224 | 37.5 | 131 | 84 | 37 | 8 | 36 | 15 |
| Gardenieae | *Brachytome hirtellata* | 154421 | 84790 | 25856 | 17919 | 37.6 | 131 | 84 | 37 | 8 | 36 | 15 |
| Gardenieae | *Heinsenia diervilleoides* | 155112 | 85353 | 25833 | 18096 | 37.6 | 131 | 84 | 37 | 8 | 36 | 15 |
| Gardenieae | *Rosenbergiodendron formosum* | 154704 | 85091 | 25791 | 18027 | 37.6 | 131 | 84 | 37 | 8 | 36 | 15 |
| Gardenieae | *Dioecrescis erythroslada* | 154704 | 85403 | 25813 | 18148 | 37.6 | 131 | 84 | 37 | 8 | 36 | 15 |
| Gardenieae | *Euclinia longiflora* | 155182 | 85364 | 25820 | 18179 | 37.5 | 131 | 84 | 37 | 8 | 36 | 15 |
| Gardenieae | *Rothmannia urcelliformis* | 154693 | 85301 | 26617 | 17374 | 37.5 | 131 | 84 | 37 | 8 | 36 | 15 |
| Gardenieae | *Rothmannia manganjae* | 155908 | 84860 | 25823 | 18189 | 37.5 | 131 | 84 | 37 | 8 | 36 | 15 |
| Gardenieae | *Gardenia jasminoides* | 154919 | 85239 | 25793 | 18093 | 37.5 | 131 | 84 | 37 | 8 | 36 | 15 |
| Gardenieae | *Gardenia sp* | 155294 | 85476 | 25847 | 18124 | 37.5 | 131 | 84 | 37 | 8 | 36 | 15 |
| Gardenieae | *Gardenia volkensii* | 155242 | 85425 | 25847 | 18124 | 37.5 | 131 | 84 | 37 | 8 | 36 | 15 |
| Gardenieae | *Feretia aeruginescens* | 129434 | 85285 | 18212 | 25937 | 36.1 | 114 | 79 | 30 | 4 | 30 | 15 |
| Gardenieae | *Schumanniophyton magnificum* | 155080 | 85384 | 25792 | 18113 | 37.4 | 131 | 84 | 37 | 8 | 36 | 12 |
| Pavetteae | *Pavetta schumanniana* | 153136 | 84643 | 25747 | 18015 | 37.5 | 131 | 84 | 37 | 8 | 36 | 15 |
| Pavetteae | *Pavetta abyssinica* | 153100 | 83586 | 25746 | 18023 | 37.5 | 131 | 84 | 37 | 8 | 36 | 15 |
| Pavetteae | *Pavetta lanceolata* | 154090 | 84579 | 25745 | 18021 | 37.5 | 131 | 84 | 37 | 8 | 36 | 15 |
| Pavetteae | *Pavetta barbertonensis* | 154151 | 83627 | 25740 | 17919 | 37.5 | 131 | 84 | 37 | 8 | 36 | 15 |
| Pavetteae | *Tarenna pavettoides* | 153786 | 84371 | 25704 | 18091 | 37.5 | 131 | 84 | 37 | 8 | 36 | 15 |
| Pavetteae | *Tarenna drummondii* | 154117 | 84431 | 25755 | 18176 | 37.5 | 131 | 84 | 37 | 8 | 36 | 15 |
| Pavetteae | *Rutidea orientalis* | 154164 | 84562 | 25744 | 18114 | 37.5 | 131 | 84 | 37 | 8 | 36 | 15 |
| Pavetteae | *Tarenna asiatica* | 154200 | 84687 | 25663 | 18098 | 37.5 | 131 | 84 | 37 | 8 | 36 | 15 |
| Pavetteae | *Tarenna mollissima* | 154252 | 84566 | 25754 | 18015 | 37.5 | 131 | 84 | 37 | 8 | 36 | 15 |
| Pavetteae | *Coptosperma supra* | 154437 | 84779 | 25756 | 18147 | 37.5 | 131 | 84 | 37 | 8 | 36 | 15 |
| Pavetteae | *Tarenna sechellensis* | 154416 | 84785 | 25755 | 18120 | 37.5 | 131 | 84 | 37 | 8 | 36 | 15 |
| Pavetteae | *Leptactina leopoldi* | 154462 | 84937 | 25655 | 18220 | 37.5 | 131 | 84 | 37 | 8 | 36 | 15 |
| Pavetteae | *Leptactina platyphylla* | 154320 | 84667 | 25768 | 18118 | 37.5 | 131 | 84 | 37 | 8 | 36 | 15 |
| Sherbournieae | *Mitriostigma greenwayi* | 153429 | 84870 | 25357 | 17845 | 37.4 | 131 | 84 | 37 | 8 | 36 | 15 |
| Sherbournieae | *Oxyanthus zanguebaricus* | 153587 | 84968 | 25363 | 17913 | 37.5 | 131 | 84 | 37 | 8 | 36 | 15 |
| Sherbournieae | *Mitriostigma axillare* | 153606 | 84955 | 25372 | 17889 | 37.3 | 131 | 84 | 37 | 8 | 36 | 15 |
| Octotropideae | *Ramosmania rodriguesi* | 155176 | 85091 | 25791 | 18032 | 37.4 | 131 | 84 | 37 | 8 | 36 | 15 |
| Octotropideae | *Burchellia bubalina* | 155176 | 85403 | 25813 | 18148 | 37.4 | 131 | 84 | 37 | 8 | 36 | 15 |
| Octotropideae | *Galiniera saxifraga* | 154723 | 84933 | 25823 | 18144 | 37.5 | 131 | 84 | 37 | 8 | 36 | 15 |
| Octotropideae | *Polyshpaeria parvifolia* | 154879 | 85075 | 25839 | 18126 | 37.5 | 131 | 84 | 37 | 8 | 36 | 15 |
| Octotropideae | *Cremaspora triflora* | 155206 | 85316 | 25845 | 18201 | 37.5 | 131 | 84 | 37 | 8 | 36 | 15 |
| Octotropideae | *Didymosalpinx norae* | 154861 | 84776 | 26076 | 17933 | 37.4 | 131 | 84 | 37 | 8 | 36 | 15 |
| Octotropideae | *Alibertia edulis* | 154508 | 84693 | 25866 | 18139 | 37.5 | 131 | 84 | 37 | 8 | 36 | 15 |
| Augusteae | *Wendlandia uvariifolia* | 154965 | 85217 | 25810 | 18127 | 37.5 | 131 | 84 | 37 | 8 | 36 | 15 |
