# Supplementary material for: Phylogenomic and comparative analyses of Coffeeae alliance (Rubiaceae): deep insights into phylogenetic relationships and plastome evolution
Source: BMC Plant Biol. 2022 Feb 26;22:88. doi: 10.1186/s12870-022-03480-5 (PMC8881883; doi:10.1186/s12870-022-03480-5)
Supplement: Supplementary file 5 — Additional file 5: Table S3. Summary of partitioning scheme for the ML tree and BI analysis. [file 12870_2022_3480_MOESM5_ESM.docx]

**Table S3.** Summary of partitioning scheme for the ML tree and BI analysis

| Matrices | Scheme lnL | Scheme AICc | Number of params | Number of sites | Number of subsets | Best Model |
| --- | --- | --- | --- | --- | --- | --- |
| protein-coding gene | -215060.25 | 430423.118 | 151 | 74430 | 1 | GTR+I+G |
| Noncoding sequences | -426186.0625 | 852674.4719 | 151 | 132477 | 1 | GTR+I+G |
| Coding sequences | -224751.2813 | 449805.1427 | 151 | 79271 | 1 | GTR+I+G |
| Complete plastomes | -676869.75 | 1354041.645 | 151 | 316,734 | 1 | GTR+I+G |
