# Supplementary material for: Phylogenomic and comparative analyses of Coffeeae alliance (Rubiaceae): deep insights into phylogenetic relationships and plastome evolution
Source: BMC Plant Biol. 2022 Feb 26;22:88. doi: 10.1186/s12870-022-03480-5 (PMC8881883; doi:10.1186/s12870-022-03480-5)
Supplement: Supplementary file 7 — Additional file 7: Table S5. Details of Relative Synonymous Codon Usage in plastome of Coffeeae alliance tribes. [file 12870_2022_3480_MOESM7_ESM.docx]

Table S5. Details of Relative Synonymous Codon Usage in plastome of Coffeeae alliance tribes

| *Species* | ENC | CBI | Codon | PCGs |
| --- | --- | --- | --- | --- |
| *Coffea sessiliflora* | 55.131 | 0.212 | 26,276 | 74827 |
| *Coffea macrocarpa* | 55.137 | 0.212 | 26,294 | 74834 |
| *Coffea stenophylla* | 55.075 | 0.213 | 26,279 | 74801 |
| *Coffea arabica* | 55.104 | 0.213 | 26282 | 74817 |
| *Coffea canephora* | 55.108 | 0.213 | 26281 | 74842 |
| *Tricalysia lasiodelphys* | 55.103 | 0.213 | 26,163 | 74709 |
| *Tricalysia pallens* | 55.106 | 0.213 | 26164 | 74709 |
| *Tricalysia hens* | 55.113 | 0.213 | 26195 | 74684 |
| *Tricalysia semidecidua* | 55.103 | 0.213 | 26163 | 74651 |
| *Belonophora coffeoides* | 55.155 | 0.212 | 26184 | 74730 |
| *Empogona ovalifolia* | 55.167 | 0.212 | 26187 | 74682 |
| *Empogona congesta* | 55.163 | 0.212 | 25183 | 74691 |
| *Feretia aeruginescens* | 55.094 | 0.213 | 22,573 | 74780 |
| *Polyshpaeria parvifolia* | 55.119 | 0.214 | 26269 | 74827 |
| *Cremaspora triflora* | 55.11 | 0.214 | 26280 | 74863 |
| *Aidia cochinchinensis* | 55.097 | 0.214 | 26273 | 74300 |
| *Aidia canthioides* | 55.107 | 0.213 | 26,292 | 74884 |
| *Alleizettella leucocarpa* | 55.107 | 0.213 | 26,295 | 74877 |
| *Diplospora dubi* | 55.12 | 0.213 | 25301 | 74877 |
| *Atractocarpus fitzalanii* | 55.129 | 0.213 | 26304 | 74860 |
| *Brachytome hirtellata* | 55.137 | 0.213 | 26282 | 74922 |
| *Catunaregam spinosa* | 55.138 | 0.213 | 26287 | 74849 |
| *Fosbergia shweliensis* | 55.135 | 0.213 | 26296 | 74867 |
| *Tarennoidea wallichii* | 55.122 | 0.213 | 26195 | 74894 |
| *Himalrandia lichiangensis* | 55.127 | 0.213 | 26299 | 74865 |
| *Duperrea pavettifolia* | 55.192 | 0.212 | 26293 | 74895 |
| *Rubovietnamia aristata* | 55.192 | 0.212 | 26284 | 74904 |
| *Rothmannia urcelliformis* | 55.2 | 0.212 | 26276 | 74843 |
| *Heinsenia diervilleoides* | 55.205 | 0.212 | 26237 | 74848 |
| *Rothmannia manganjae* | 55.065 | 0.216 | 26274 | 74883 |
| *Gardenia volkensii* | 55.112 | 0.213 | 26281 | 75668 |
| *Gardenia sp* | 55.105 | 0.213 | 26280 | 74836 |
| *Gardenia jasminoides* | 55.112 | 0.213 | 26281 | 74849 |
| *Dioecrescis erythroslada* | 55.104 | 0.214 | 26225 | 74865 |
| *Rosenbergiodendron formosum* | 55.104 | 0.214 | 26224 | 74820 |
| *Euclinia longiflora* | 55.096 | 0.215 | 25784 | 74820 |
| *Burchellia bubalina* | 55.168 | 0.213 | 26273 | 74878 |
| *Tarenna sechelleensis* | 55.168 | 0.213 | 26057 | 74873 |
| *Ramosmania rodriguesi* | 55.139 | 0.215 | 26272 | 74872 |
| *Coptosperma supra* | 55.191 | 0.212 | 26262 | 74915 |
| *Tarenna asiatica* | 55.148 | 0.214 | 26261 | 74341 |
| *Pavetta abyssinica* | 55.101 | 0.214 | 26208 | 74794 |
| *Pavetta schumanniana* | 55.101 | 0.214 | 26207 | 74786 |
| *Pavetta lanceolata* | 55.11 | 0.214 | 26208 | 74832 |
| *Pavetta barbertonensis* | 55.106 | 0.214 | 26205 | 74700 |
| *Tarenna pavettoides* | 55.112 | 0.214 | 26266 | 74664 |
| *Tarenna drummondii* | 55.106 | 0.214 | 26275 | 74875 |
| *Leptactina platyphylla* | 55.13 | 0.214 | 26259 | 74822 |
| *Leptactina leopoldi* | 55.162 | 0.214 | 26262 | 74714 |
| *Rutidea orientalis* | 55.085 | 0.215 | 26258 | 74759 |
| *Tarenna mollissima* | 55.109 | 0.215 | 26049 | 74274 |
| *Galiniera saxifraga* | 55.116 | 0.214 | 26300 | 74971 |
| *Bertiera iturensis* | 55.189 | 0.212 | 26137 | 74629 |
| *Bertiera breviflora* | 55.158 | 0.213 | 25862 | 74226 |
| *Bertiera laxa* | 55.22 | 0.211 | 25873 | 74261 |
| *Didymosalpinx norae* | 55.011 | 0.215 | 25789 | 75230 |
| *Mitriostigma greenwayi* | 55.132 | 0.214 | 26047 | 74461 |
| *Mitriostigma axillare* | 55.164 | 0.213 | 26052 | 74534 |
| *Oxyanthus zanguebaricus* | 55.129 | 0.214 | 26065 | 74547 |
| *Wendlandia uvariifolia* | 55.23 | 0.211 | 26266 | 74818 |
| *Schumanniophyton magnificum* | 55.238 | 0.21 | 26243 | 75231 |
| *Alibertia edulis* | 55.139 | 0.215 | 26295 | 74915 |
