# Supplementary material for: Phylogenomic and comparative analyses of Coffeeae alliance (Rubiaceae): deep insights into phylogenetic relationships and plastome evolution
Source: BMC Plant Biol. 2022 Feb 26;22:88. doi: 10.1186/s12870-022-03480-5 (PMC8881883; doi:10.1186/s12870-022-03480-5)

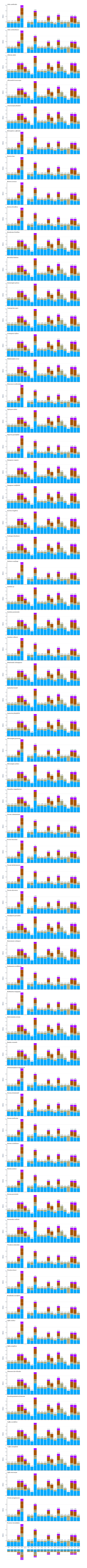

Supplement: Supplementary file 2 — Additional file 2. The codon usage bias in all the protein-coding genes of the plastome of the 62 Coffeeae allinces tribes. [file 12870_2022_3480_MOESM2_ESM.pdf]
